# A Dedicated Nephrology Daycare Unit: A Step Toward a Patient-Centered Approach

**DOI:** 10.7759/cureus.67324

**Published:** 2024-08-20

**Authors:** Ghada Ankawi

**Affiliations:** 1 Department of medicine, Division of Nephrology, King Abdulaziz University Faculty of Medicine, Jeddah, SAU

**Keywords:** immunosuppression, renal biopsy, dialysis access, day-admission, daycare

## Abstract

Background

Nephrology services encompass a wide range of day-care procedures. Securing beds for day admissions can be challenging and may lead to significant delays in patient management.

Objective

This study aims to describe the impact of establishing a dedicated nephrology daycare unit at a tertiary care center.

Methods

Since January 2021, a dedicated nephrology daycare unit has been operational at King Abdulaziz University Hospital (KAUH) in Jeddah, Saudi Arabia. This observational study retrospectively reviewed the admission records to the KAUH nephrology daycare unit from January 2021 to December 2023. Admissions with missing data were excluded from the analysis. The study outcomes included: 1. the number of patients served in the unit, 2. the scope of services provided, 3. the “time to completion” of immunosuppressive therapy administered in the unit, and 4. the rate of complications related to admission to the unit.

Results

There were 233 admissions for 157 patients. The scope of procedures included: 1. administration of immunosuppressive therapy (42 doses of cyclophosphamide, 70 doses of rituximab, three doses of methylprednisolone), 2. renal biopsies (25 procedures), 3. tunneled dialysis catheter procedures (40 procedures, both insertion and removal), 4. dialysis access angioplasty (three procedures), 5. IV iron therapy (45 admissions), and 6. other miscellaneous causes (five admissions). Ideal time to completion of cyclophosphamide therapy was achieved in 86% of patients, with the remaining 14% experiencing delays due to reasons other than bed availability. Time to completion of rituximab therapy was achieved without delay in 85% of patients, with a time interval of less than 21 days. There were no reported complications associated with admission to the unit.

Conclusions

Establishing a dedicated nephrology daycare unit facilitates the delivery of nephrology day-procedures and reduces delays in therapy.

## Introduction

Chronic kidney disease (CKD) is defined as kidney damage or decreased kidney function persisting for three or more months [1]. CKD is an increasing public health concern, with an estimated global prevalence of 9.1% (8.5 to 9.8) among adults between 1990 and 2017 [2]. Additionally, in 2019, there was a notable increase in CKD-related deaths by 10.1% and in disability-adjusted life years (DALYs) by 81.7% compared to the 1990s [3]. It is estimated that more than 850 million people worldwide are affected by kidney disease in the form of acute kidney injury, CKD, or requirement of renal replacement therapy [4]. Therefore, measures to facilitate early detection, accurate diagnosis, and management of kidney disease are of utmost importance.

Many diagnostic workups and therapeutic interventions require day admission to the hospital. Indications for day admission include performing renal biopsies, administering immunosuppressive therapy, and conducting dialysis access-related procedures such as insertion, removal, and angioplasty. A significant cause of delays in diagnosing the underlying etiology of CKD and implementing appropriate management is the availability of beds. Here, we aim to describe the impact of establishing a dedicated nephrology daycare unit on facilitating nephrology-related day admissions.

## Materials and methods

This is a retrospective observational study that includes admissions to the nephrology daycare unit at a tertiary care center. The inclusion criteria encompassed all admissions from January 2021 to December 2023, while the exclusion criteria were admissions with missing data. The study outcomes were: 1. the number of patients served in the unit, 2. the scope of services provided, 3. the "time to completion" of immunosuppressive therapy administered in the unit, defined as the time interval between the first and last dose, and 4. the rate of complications related to admission to the unit. Data were collected from the unit's admission records.

The nephrology daycare unit shares the same reception area as the dialysis unit. A dedicated team of nurses and physicians staffs the unit based on a monthly schedule. Patients are assessed and managed by both nurses and physicians according to the unit's protocols for various admission indications. Our unit is designed to offer specialized and immediate care to patients with kidney-related ailments, facilitating procedures that traditionally required longer hospital stays. The primary outcomes measured in this study were multifaceted. Firstly, the number of patients served provided a quantitative measure of the unit's capacity and reach within the community. Secondly, the scope of services rendered illustrated the unit's versatility and ability to address a variety of nephrological needs, from routine procedures to more complex therapeutic interventions. Thirdly, the "time to completion" of immunosuppressive therapy was a critical metric, reflecting the efficiency and effectiveness of treatment protocols within the unit, which is vital for managing conditions that require prompt and precise medical attention. Lastly, monitoring the rate of complications associated with admissions offered insights into the safety and quality of care provided, helping to identify potential areas for improvement.

## Results

**Figure 1:**
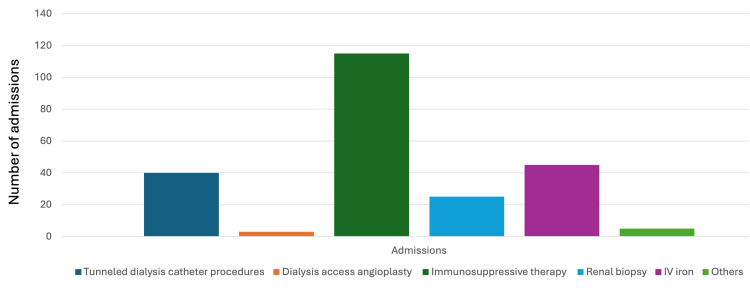
Scope of service

There were 233 admissions for 157 adult patients. The scope of services included: 1. administration of immunosuppressive therapy (42 doses of cyclophosphamide, 70 doses of rituximab, three doses of Methylprednisolone), 2. renal biopsies (25 procedures), 3. tunneled dialysis catheter procedures (40 procedures, both insertion and removal), 4. dialysis access angioplasty (three procedures), 5. Intravenous iron therapy (45 admissions), and 6. other miscellaneous causes (five admissions) (shown in Fig. 1).

Regarding the "time to completion" of immunosuppressive therapy, the most commonly used agents in our unit are cyclophosphamide, used as an induction therapy for lupus nephritis, and rituximab, used as a therapeutic agent for glomerular disease.

**Figure 2:**
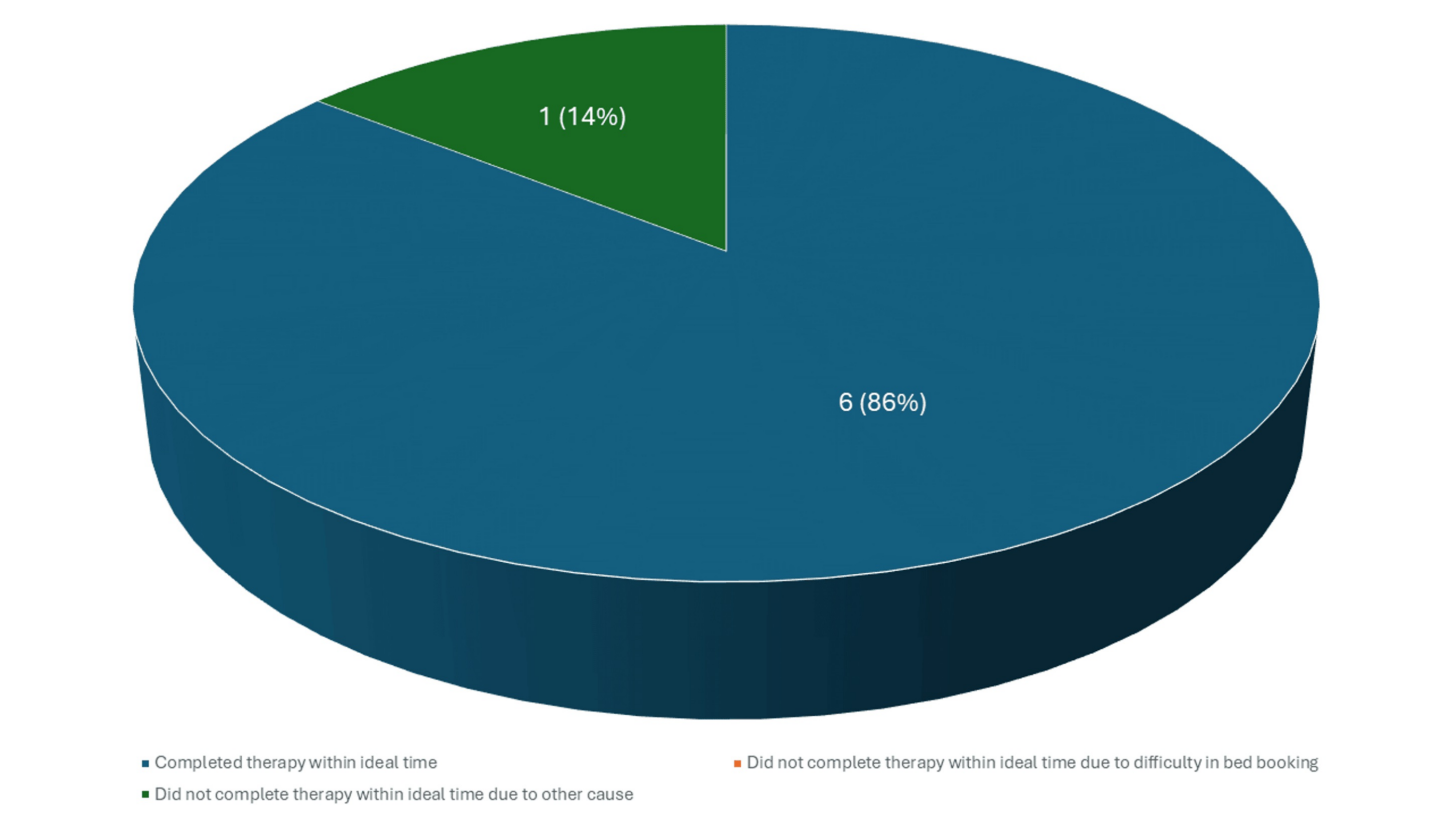
Time to completion of cyclophosphamide therapy

Cyclophosphamide, as an induction therapy for lupus nephritis, is typically given in six doses within a 3-month period [5]. Often, the first dose is administered on an inpatient basis, and patients are scheduled to receive the remaining five doses on an outpatient basis. Therefore, admissions of patients who received five or six doses were examined. A total of 11 patients received cyclophosphamide during the study period, of whom seven had five or six doses. Among these seven patients, six (86%) completed the therapy within three months (shown in Fig. 2).

**Figure 3:**
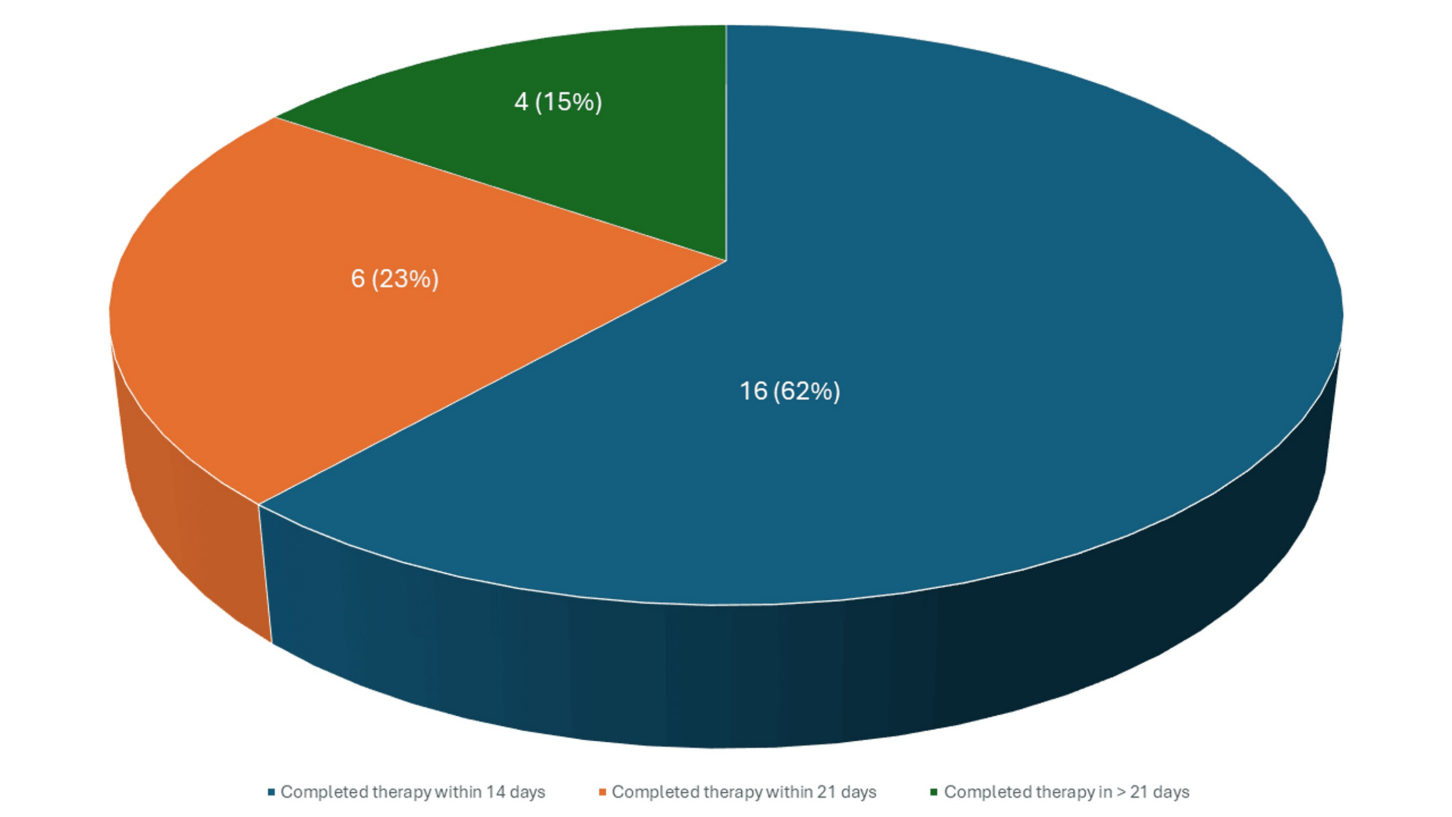
Time to completion of rituximab therapy

The delay for the one patient who did not complete the therapy on time was not due to difficulty in booking the required bed, but rather to an active infection that prevented her from receiving the scheduled dose. As for rituximab, the typical indication for administration in our unit is the treatment of glomerular disease, particularly minimal change disease and focal segmental glomerulosclerosis. The doses are typically given as two doses 14 days apart; however, maintenance regimens may be administered every six months. A total of 40 patients received rituximab during the study period. We excluded 14 patients from the analysis as three received rituximab as maintenance therapy and 11 received a single dose. Among the remaining 26 patient admissions that were included, the time intervals between the start and completion of therapy were as follows: therapy was completed in less than 14 days in 16 out of 26 (62%), which is ideal; therapy was completed in more than 14 but less than 21 days in six out of 26 (23%), which is acceptable; and therapy was completed in more than 21 days in four out of 26 (15%), which is suboptimal (shown in Fig. 3).

Finally, no complications were directly related to admission to the nephrology daycare unit. Patients who developed complications related to the procedures performed, such as hematuria following a renal biopsy, were managed according to standard protocol by admitting them to the inpatient service for observation and further management.

## Discussion

Glomerulonephritis (GN) is a reversible cause of kidney disease that, if identified and treated promptly, can prevent progression to end-stage kidney disease (ESKD). In the United States, GN contributes to 10% to 15% of cases of ESKD, ranking it as the third most common cause following diabetes mellitus and hypertension [6, 7]. Kidney biopsy is crucial for diagnosing GN [8] and unexplained acute kidney injury. Kidney biopsies should be performed promptly as delay in diagnosis and therapy is associated with poor outcomes [9, 10, 11]. Barriers to performing biopsies, such as bed availability, have been significant in our hospital. One of the primary goals of establishing a dedicated nephrology daycare unit was to ensure timely access to beds for kidney biopsies, reducing wait times. Previously, the average wait time for a bed in our hospital was up to four weeks. Since the unit's establishment, kidney biopsies are typically performed within a week. Patients are typically admitted to the nephrology daycare unit early in the morning, where they are assessed by the nephrology team. They are then sent to the interventional radiology suite for their procedure. Afterward, they are monitored in the nephrology daycare unit until the end of the day to ensure stability before being discharged. Moreover, once a diagnosis is confirmed, patients often require parenteral therapies such as cyclophosphamide and rituximab, as previously mentioned. Administering these therapies has historically been challenging in our hospital. For instance, the cyclophosphamide Euro-lupus regimen, typically administered over three months, has sometimes been extended to four or five months in the past. Establishing the dedicated unit has significantly improved the timeliness of completing these therapies.

On the other end of the spectrum, patients who progress to ESKD, whether due to GN or other causes, often require access to a day admission unit for dialysis access-related procedures [12]. Complications related to access, such as infection, thrombosis, and blockage, require prompt intervention [13]. For example, timely removal of infected lines is crucial for source control and treating sepsis [14]. At our institution, removing infected tunneled dialysis catheters was challenging, even for hospitalized patients, as it required operating room booking and involvement from the vascular surgery or interventional radiology teams, leading to significant delays. The creation of our unit, along with its protocol for bedside tunneled catheter removals by nephrologists, has greatly facilitated the timely removal of infected catheters. After the procedure, patients are typically observed in the nephrology unit by the nephrology team before being transferred back to the inpatient ward. Establishing the unit facilitated the timely management of dialysis catheter complications, especially concerning the removal of infected lines in cases of dialysis catheter-related bacteremia. Therefore, the second most common reason for admission to the unit was dialysis access-related procedures. Additional indications for admission to a nephrology daycare unit include peritoneal dialysis (PD)-related procedures such as PD catheter insertion and removal. However, our unit has not yet expanded to include these indications.

While the benefits mentioned can be achieved by daycare units not exclusively dedicated to nephrology patients, our experience suggests that dedicated units offer additional advantages beyond expediting procedure bookings. These include enhanced continuity of care, expanded opportunities for patient education, and strengthened rapport between the nephrology team (physicians and nurses) and patients. Improved relationships contribute significantly to better patient adherence to management plans [15].

Our study has several limitations. First, all the data were gathered from unit admission records without access to individual patient records, which limited the data available for analysis. However, our main focus is on the impact of establishing the unit on our typical indications for admission, not on patient outcomes. Second, data describing study outcomes prior to establishing the unit were not available and therefore were not examined for comparison. This would have strengthened our results and documented improvements in study outcomes more accurately. Despite these limitations, our study aims to provoke thought and represents a step in the long journey toward patient-centered care. 

## Conclusions

Our study highlights the efforts we can make to enhance the patient experience, despite its inherent limitations. While direct access to patient records would have provided more comprehensive data, our focus on the impact of establishing the unit on admission indications remains valuable. Specifically, the impact of early appointment times in facilitating early diagnosis and timely management is noteworthy.

For future research, including historical data for comparison could strengthen conclusions further. Nevertheless, our study contributes to a broader understanding of how specialized units like ours positively influence healthcare delivery. This insight can pave the way for advancements in patient-centered care within nephrology and beyond. In today's era of complex medical care, we advocate for ongoing efforts to enhance patient experiences and uphold a patient-centered care approach.
